# Endoscopic management of refractory gastrointestinal non-variceal bleeding using Histoacryl (*N*-butyl-2-cyanoacrylate) glue

**DOI:** 10.1093/gastro/gov019

**Published:** 2015-05-19

**Authors:** Damien CK Loh, Robert B Wilson

**Affiliations:** Department of Upper Gastrointestinal Surgery, Liverpool Hospital, Sydney, Australia

**Keywords:** Histoacryl glue, N-butyl-2-cyanoacrylate, endoscopy, non-variceal upper gastrointestinal bleeding

## Abstract

**Background:** Histoacryl glue (N-butyl-2-cyanoacrylate) has well-established utility in the endoscopic management of gastrointestinal variceal bleeding. The role of Histoacryl glue in non-variceal bleeding is less clear, and there are few articles describing its use in this setting.

**Methods:** Six patients with intractable non-variceal gastrointestinal bleeding were managed using injection of Histoacryl glue. All patients had previously failed conventional endostasis and/or interventional angioembolization and were not suitable for emergency salvage surgery due to serious comorbidities or unacceptable anaesthetic risk. An endoscopic Lipiodol-Histoacryl-Lipiodol sandwich injection technique was used in these patients. The clinical outcomes and complications were evaluated.

**Results:** There were four females and two males with a mean age of 55 years. Bleeding lesions included gastric ulcers (*n* = 2), duodenal ulcers (*n* = 2), duodenal gastrointestinal stromal tumor (GIST) (*n* = 1) and rectal ulcers (*n* = 1). All patients had successful Histoacryl endostasis without the requirement for salvage surgery. There was no treatment-related morbidity and no mortality. Two patients had further bleeding after initial Histoacryl endostasis, which was successfully controlled with further endoscopic Histoacryl injection.

**Conclusion:** Histoacryl endostasis should be included in the treatment algorithm for refractory non-variceal gastrointestinal bleeding.

## Introduction

Cyanoacrylate (Histoacryl glue; N-butyl-2-cyanoacrylate; B. Braun, Melsungen, Germany) is a liquid tissue adhesive that has well-established utility in the endoscopic management of gastrointestinal variceal bleeding [1–4]. However, the role of Histoacryl glue in non-variceal bleeding is less clear [5–11]. This is due to the emergence of alternative modes of endostasis, transarterial embolization (TAE) and potential side effects of endoscopic Histoacryl use in peptic ulcer disease.

The incidence of complications and comparative safety profiles are important factors to consider in the choice of a therapeutic procedure. Despite the use of Histoacryl glue in gastric variceal bleeding being relatively safe, there are still potential serious complications. The overall morbidity from a series of 753 patients with gastric varices treated with endoscopic Histoacryl glue, as reported by Cheng *et al*, was 6.77%, and the treatment-related mortality was 0.53%. Thirty-three patients (4.38%) experienced rebleeding related to early glue cast extrusion. Distant embolization occurred in five cases (0.7%), and sepsis occurred in 10 (1.33%) [4].

Concerns about the use of Histoacryl glue in non-variceal bleeding means it is usually reserved for salvage endostasis. Any comparison with salvage TAE or surgery in non-variceal bleeding requires a fundamental understanding of the potential risks of Histoacryl endostasis. We present our experience with Histoacryl endostasis and a comprehensive review of current treatment options in patients with refractory non-variceal gastrointestinal bleeding.

## Methods

Six patients were managed with injection of Histoacryl glue by a single surgeon experienced in therapeutic endoscopy. The institution is a tertiary referral hospital with a dedicated upper GI bleeding roster run by the gastroenterology department. All patients in this series were referred to the upper GI surgery unit after failure of conventional treatments for acute gastrointestinal bleeding, including three patients who had both endoscopic management and TAE prior to referral. Standard therapy included high-dose IV proton pump inhibition and *Helicobacter pylori* eradication when indicated but also sucralfate, vitamin C and *N*-acetyl cysteine administration in two patients.

After clinical evaluation of the six patients, Histoacryl endostasis was considered safer than emergency surgery because of significant patient comorbidities and unacceptable anaesthetic risk. A standard gastroscope, Luer lock syringes, foot-operated water irrigation pump and eye protection for staff were used. The endoscopy channel and injecting catheter were primed with plain Lipiodol. An endoscopic Lipiodol-Histoacryl-Lipiodol ‘sandwich’ or ‘push’ injection technique was used in all patients, with aliquots of 1 ml volume of 0.5 ml Lipiodol/ 0.5 ml Histoacryl injected directly into the bleeding site. The catheter was flushed with 0.8–1.0 ml plain Lipiodol after the glue mixture was injected to deliver the entire glue mixture, and the needle was withdrawn from the lesion. This prevents the needle from being glued into the lesion, which can potentially occur with undiluted glue or with a distilled water flush, which increases glue polymerization. Minimizing the volume of glue/flush injected may decrease the risk of embolization [1–11]. Further injections of glue mixture (1 ml) were used if the initial attempt was unsatisfactory, or for treatment of multiple gastric ulcers.

A 24-hour emergency endoscopy and interventional radiology service is provided. Patients were resuscitated in the gastroenterology ward or Intensive Care Unit (ICU) before being transported to endoscopy, operating theatres or interventional radiology. Most patients were intubated for their endoscopy due to the risk of aspiration or comorbidities. Patients were managed in the high dependency unit (HDU)/ICU post procedure and monitored for signs of rebleeding or complications. Patients were managed by the surgical unit until discharge from hospital.

## Results

Table 1 summarizes our series of six cases in which injection of Histoacryl glue was used for salvage control of non-variceal gastrointestinal bleeding. There were four females and two males with a mean age of 55 years. Bleeding lesions included gastric ulcers (*n* = 2), duodenal ulcers (*n* = 2), duodenal GIST (*n* = 1) and rectal ulcers (*n* = 1).

**Table 1.** Case summary

	Case 1	Case 2	Case 3	Case 4	Case 5	Case 6
**Age (years)**	67	63	45	55	39	60
**Sex**	Female	Male	Male	Female	Female	Female
**Co-morbidities**	Type 2 diabetes, *S aureus* septicaemia, Acute kidney injury, Acute myocardial infarction, Acute respiratory distress syndrome, Coagulopathy, Atrial fibrillation, Fractured R ala/L1, Pneumonia, NSAID peptic ulcer disease	Type 2 diabetes, Ischaemic heart disease, Fractured neck of femur, NSAID-induced acute renal failure (requiring dialysis), Peripheral vascular disease, Pulmonary embolus/IVC filter, Morbid obesity, NSAID peptic ulcer disease	Alcoholic cirrhosis, Portal hypertension NSAID peptic ulcer disease	Hyperlipidaemia	None	Cytomegalovirus colitis, Multiple myeloma
**Shock**	Yes	Yes	Yes	Yes	Yes	Yes
**Pulse**	82	110	125	105	128	
**Systolic blood pressure (mmHg)**	91	120	143	101	116	
**Haemoglobin (g/L)**	78	52	72	89	88	
**Albumin (g/L)**	27	24	18	25	21	
**Urea (mmol/L)**	15.8	55.1	11.1	16.2	13.6	
**Melaena**	Yes	Yes	No	Yes	No	
**Syncope**	No	No	No	No	Yes	
**Endoscopy findings**	Duodenal ulcers	Gastric antral ulcerLesser curve ulcers	Gastric antral ulcers	Duodenal ulcer	Duodenal tumour	Rectal ulcer
**Forrest type**	Ib	IIb	IIa	Ia	III	N/A
**Ulcer size (mm)**	<10	40	20	7	<10	20
**Evidence of recent bleeding**	Yes	Yes	No	Yes	Yes	Yes
**Exposed vessel >2mm**	Yes	No	No	No	No	
**Diagnosis**	NSAID ulcer	NSAID ulcer	NSAID ulcer	Benign ulcer	GIST	Benign rectal ulcer
**Initial endoscopic management**	None	None	None	Adrenaline + endoclips	None	None
**TAE Vessel embolized**	Yes Gastroduodenal artery branch, Coeliac artery branch	Yes Left gastric artery, Left gastric artery territory	No	No	No	Yes Anterior rectal artery
**In-hospital rebleed**	Yes	Yes	Yes	Yes	Yes	Yes
**Repeat endoscopic management**	Histoacryl	Adrenaline + heater probe Histoacryl	Histoacryl	Histoacryl	Histoacryl	Histoacryl
**Blatchford score[****12****]**	15	16	13	13	13	Nil
**Rockall score[****13****]**	8+	6	7	4	2	Nil
**Rockall predicted rebleeding risk [****14****]**	41.8%	32.9%	43.8%	14.1%	5.3%	Nil
**Rockall predicted mortality [****14****]**	41.1%	17.3%	27.0%	5.3%	0.2%	Nil
**Endoscopic follow-up**	N/A	Complete healing	N/A	Distal gastrectomy post stabilization	Whipple’s resection post staging and stabilization	Complete healing

IVC, inferior vena cava; NSAID: non-steroidal anti-inflammatory drug; N/A: not applicable

**Figure 1.**
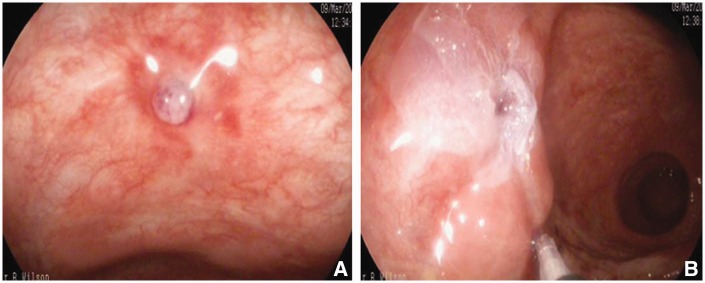
Histoacryl glue injection for actively bleeding duodenal ulcer (Case 1). (A) Duodenal ulcer with visible vessel. (B) Duodenal ulcer post Histoacryl glue injection.

**Figure 2.**
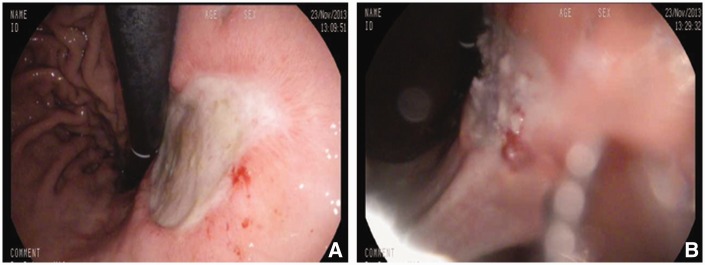
Histoacryl glue injection for large gastric antral ulcers (Case 2). (A) Lesser curve ulcer with a clean base. (B) Lesser curve ulcer post Histoacryl glue injection.

All patients had successful Histoacryl endostasis (Figures 1 and 2) without the requirement for salvage surgery. There was no treatment-related morbidity due to Histoacryl injection and no mortality. Two patients had further bleeding after initial Histoacryl endostasis, which was successfully controlled with further endoscopic Histoacryl injection. No patients required re-admission for bleeding or treatment-related complications after being discharged from hospital. The Rockall predicted mortality (based on conventional treatments) in our case series was substantial compared with the observed mortality with Histoacryl endostasis, which was zero (Table 1).

## Discussion

Histoacryl polymerizes rapidly with an exothermic reaction on contact with anions in blood, serum or tissues or hydroxyl anions in water. It forms a solid, stable, inflexible glass-like polymer that can be subsequently extruded. Extrusion of the glue cast can occur between one week and six months after injection into gastric varices. Cyanoacrylate toxicity is in part related to the formaldehyde, cyanoacetate and heat generated (88°C) during polymerization. When injected, it causes an intense acute inflammatory reaction in the vessel wall and surrounding perivascular tissues that can lead to endothelial damage and potential tissue necrosis, perforation or fistula formation. Mixing with iodinated poppy seed oil (Lipiodol; Guerbert, Roissy, France) prolongs the polymerization time, increases the viscosity and provides suitable radio-opacity. Use of water to flush the endoscope catheter during injection will activate polymerization [1, 15]. Preparation of the endoscope channels and catheters with Lipiodol, mandatory use of Luer lock syringes and eye protection will prevent damage to endoscopes and injury to staff.

Despite these potential side effects, Histoacryl is used widely in the UK, European, Australian and Asian hospitals for treatment of gastric varices. The US FDA has limited its use in the USA due to the rare but serious problem of thromboembolism [9]. In 2000 Lee *et al* published the only randomized controlled trial of hypertonic saline/epinephrine (HSE) monotherapy versus Histoacryl injection in bleeding peptic ulcers. The rebleeding rate in ulcers with visible arterial bleeding was 42% in the HSE group and 14% in the Histoacryl group (*P* = 0.039). There were no reported complications in the HSE group, but two patients (3.3%) in the Histoacryl cohort developed arterial embolization with infarction, one of whom died [10]. Both patients had active arterial bleeding from chronic gastric ulcers. Because of this risk of embolization, it was suggested that the use of less-diluted Histoacryl seemed preferable in the treatment of bleeding peptic ulcer. This is of particular relevance when treating large bleeding proximal gastric ulcers, due to proximity of the coeliac trifurcation.

With these safety concerns and continued development of coaptive thermal and mechanical devices, routine Histoacryl endostasis in non-variceal upper gastrointestinal bleeding (NVUGIB) has declined. However, its successful use as rescue therapy for patients with refractory NVUGIB from ulcers, tumours or Dieulafoy’s lesions has been reported in small non-randomized series since 2002 [5–11]. Our present series of six patients also showed that Histoacryl can provide a safe and definitive alternative to urgent or emergency surgery in high-risk patients when conventional endostasis techniques have failed.

Durable conventional endostasis rates in NVUGIB vary from 82–96% [14, 16–22]. Most patients will be managed with initial endoscopy and attempted haemorrhage control with saline/adrenaline injection combined with endoclip or heater probe application [18]. However, the deep posterior duodenal ulcer with involvement of the gastroduodenal artery or the large (>2 cm) lesser curve gastric ulcer are not always well controlled with the above endostasis techniques [19]. Other independent risk factors for intractable endostasis in peptic ulcers include age ≥70 years, shock on hospital admission, hemoglobin <8.0 mg/dL, serum albumin <33 g/L, exposed vessels ≥2 mm diameter in the ulcer base and Forrest-type Ia and Ib lesions [23]. Rockall *et al* identified patient-specific risk factors associated with a high risk of rebleeding including heart failure, ischaemic heart disease and renal failure [13]. The Glasgow Blatchford Score (GBS) has been shown to be equivalent to the full Rockall score in predicting the need for endoscopic/surgical intervention and mortality in upper gastrointestinal haemorrhage[12, 14, 24].

The choice of subsequent therapy in intractable bleeding includes re-endoscopy, TAE or urgent surgery. Rebleeding after initial endotherapy can be controlled in 75% of patients with a second endoscopy, which is safer than going to surgery [17]. Technical success and clinical success rates of TAE are quoted as 91–100% and 61–100%, respectively. However, the discrepancy between technical and clinical success rates should be noted [25, 26]. A recent pooled analysis of the six retrospective series of TAE versus surgery after failed endostasis revealed a better treatment-related complication rate in the TAE versus surgery groups (20–54% *vs* 37–68%), a high but similar mortality rate (18.7% *vs* 23.7%; *P* = 0.49) and a substantially higher risk of rebleeding after TAE versus surgery (28.6% *vs* 14.9%; *P* = 0.002) [18, 19]. The problem of rebleeding after technically successful TAE, which occurred in three of our patients, provides a management challenge. Such bleeding can occur if only coils, rather than additional gelfoam, are used during TAE or if collaterals from the right gastric or inferior pancreaticoduodenal arteries subsequently open after prior occlusion of the left gastric or gastroduodenal arteries [25]. This may explain the failure of TAE in cases 1 and 2 in our series. A randomized, controlled multi-centre trial (NCT 00766961) in Hong Kong comparing salvage surgery versus TAE is due for completion in 2017 [18]. This may determine if surgery for bleeding duodenal ulcer is indeed dead [25].

A cooperative approach between treating gastroenterologists and upper GI surgeons with respect to risk assessment, resuscitation requirements, early notification of high-risk patients, pooling of resources and appropriate institutional treatment algorithms is important in the management of NVUGIB [16, 19, 20]. Surgeons are rarely required to intervene in NVUGIB due to the success of primary endoscopic management. However, the patients they are asked to salvage can be elderly, malnourished, deconditioned, comorbid, shocked, hypothermic, cirrhotic, coagulopathic, taking antiplatelet agents or have varying degrees of multi-organ failure with poor physiological reserve. Prior endoscopic therapy also selects a high-risk group of patients with large chronic bleeding ulcers that may require antrectomy and drainage rather than simple oversewing [19, 21]. This is why urgent surgery for recurrent in-hospital bleeding is associated with such high morbidity and increasing mortality rates. For example, the incidence of salvage surgery for bleeding peptic ulcer in the UK National Audit in 1993–1994 was 12%, with a mortality of 24%; in the latest audit from 2007, salvage surgery was required in 2% of patients with acute upper gastrointestinal bleeding with a mortality of 29% [16, 19].

Histoacryl glue endostasis, when administered by a suitably experienced endoscopist, can be a safe, inexpensive and effective salvage alternative to surgery when other measures have failed, or if TAE is not available [5–11, 22]. Prospective randomized trials comparing Histoacryl endostasis with TAE or salvage surgery would further delineate the treatment algorithm for non-variceal gastrointestinal bleeding.

*Conflict of interest statement*: none declared.
